# Maternal body condition during late-pregnancy is associated with in utero development and neonatal growth of Holstein calves

**DOI:** 10.1186/s40104-021-00566-2

**Published:** 2021-04-02

**Authors:** A. S. Alharthi, D. N. Coleman, I. A. Alhidary, M. M. Abdelrahman, E. Trevisi, J. J. Loor

**Affiliations:** 1 Department of Animal Production, College of Food and Agriculture Sciences, King Saud University, Riyadh, 11451 Saudi Arabia; 2 Department of Animal Sciences and Division of Nutritional Sciences, University of Illinois, Urbana, IL 61801 USA; 3 Department of Animal Sciences, Food and Nutrition (DIANA), Faculty of Agriculture, Food and Environmental Science, Università Cattolica del Sacro Cuore, 29122 Piacenza, Italy

**Keywords:** Metabolism, Neonate, Nutritional programming, Transition cow

## Abstract

**Background:**

Nutritional management in the dry period can alter body condition score (BCS) in dairy cows, a subjective measure of body fat. As such, differences in BCS during late-pregnancy not only mirror nutrient utilization by fat depots, but also can play important roles on the metabolic and hormonal environment. We investigated the association between cow BCS during late-pregnancy on developmental parameters and blood variables of neonatal calves. Forty-nine multiparous Holstein cows were retrospectively divided by prepartal BCS into normal BCS ≤3.25 (NormBCS; 3.02 ± 0.17, *n* = 30) or high BCS ≥3.75 (HighBCS; 3.83 ± 0.15, *n* = 19) groups. Plasma samples were collected from cows at − 10 d relative to parturition. Body weight, hip and wither height, hip width and body length were measured at birth and weekly through weaning (42 d of age) and until 9 weeks of age. Calf blood samples were collected from the jugular vein at birth (before receiving colostrum, 0 d), 24 h after first colostrum and at 7, 21, 42 and 50 d of age. The data were subjected to ANOVA using the mixed procedure of SAS. The statistical model included day, BCS, and their interactions.

**Results:**

Dry matter intake (kg/d or % of body weight) during the last 4 weeks of pregnancy was lower (*P* ≤ 0.06) in HighBCS cows. Plasma concentrations of fatty acids, ceruloplasmin, and nitric oxide were greater overall (*P* < 0.05) at d − 10 prior to calving in HighBCS cows, and they tended (*P* = 0.08) to have greater concentrations of reactive oxygen metabolites. Birth body weight was lower (*P* = 0.03) in calves born to dams with HighBCS. In addition, plasma concentrations of fatty acids, albumin and urea (*P* < 0.05) were greater in those calves. Although calves born to cows with HighBCS maintained a lower postnatal body weight (*P* = 0.04), hip and wither height, hip width, and body length, there was no difference (*P* > 0.05) in daily starter intake and average daily gain due to maternal BCS.

**Conclusions:**

Overall, results highlight an association between BCS during late-gestation on in utero calf development and postnatal growth. A high maternal BCS during late-gestation was associated with lower calf body weights, which could be due to lower maternal intakes and a state of inflammation and metabolic stress.

**Supplementary Information:**

The online version contains supplementary material available at 10.1186/s40104-021-00566-2.

## Background

Maintenance of dairy cow body condition score (BCS) during the periparturient period and its relation to health and production is an ongoing area of research. Cows that calve at a high BCS during this period (classified as a score ≥ 3.5) tend to have lower intakes and milk yield and mobilize more adipose tissue [1]. Furthermore, cows with high BCS have greater levels of inflammation and oxidative stress variables, which increase the risk of developing metabolic disorders such as ketosis and fatty liver [1, 2]. Much of the research on BCS during the dry period and early lactation has focused on its relationship to cow performance, lipid metabolism, inflammation, metabolic disorders and management practices to maintain BCS [3–5]. However, the role of maternal BCS on fetal growth during late-pregnancy and the neonatal period in dairy cattle is not fully understood.

In non-ruminants, it is well known that maternal diet, health and lifestyle are important factors that can affect fetal development and subsequent health, development and performance of the offspring [6]. Of these maternal factors, there is abundant evidence that maternal body mass in humans alters offspring growth, metabolism and health [7]. In particular, increases in maternal body mass are associated with greater maternal inflammation and oxidative stress status, both of which can program offspring towards greater inflammatory responses [8]. Maternal stress (e.g. heat stress, restricted energy intake) during late-gestation in dairy cattle affects immune function and production of the offspring, with effects potentially extending through the first lactation in the heifers [9, 10].

Late-gestation represents an important period in fetal development due to the rapidly increasing nutrient needs of the fetus. During this period in dairy cattle, circulating nutrients and blood variables can directly alter placental and fetal metabolism [11], rendering the maintenance of maternal body condition in late-gestation an important factor in modulating fetal development. While this has been well-studied in non-ruminants, there is a paucity of information on the effects of maternal BCS on the newborn calf.

Current recommendations for dry cow management suggest feeding higher-energy diets during the last 3–4 weeks of gestation to support the rapid increase of fetal growth, and to prepare the cow for the sudden decrease in dry matter intake (DMI) around parturition [12]. As in non-ruminants, the survival and growth of newborn calves may be affected by nutrient supply during the prepartal period [13]. For instance, nutrient restriction during late-gestation reduced beef and dairy calf growth [14, 15]. Additionally, one study in dairy cows reported that high maternal BCS during gestation was associated with a small decrease in daughter milk yield [16]. However, alterations in birth weights or postnatal growth and metabolism were not assessed.

We hypothesized that calf growth and performance during postnatal life would be affected by maternal BCS prior to calving. To address the hypothesis, measures of growth and development were collected along with plasma concentrations of biomarkers related to energy metabolism, liver function, inflammation and oxidative stress throughout the postnatal period.

## Methods

All procedures for this study were conducted in accordance with a protocol approved by the Institutional Animal Care and Use Committee (IACUC) of the University of Illinois (protocol #17168). Forty-nine multiparous Holstein cows were retrospectively divided by prepartal BCS at − 4 weeks relative to parturition into normal BCS ≤ 3.25 (*n* = 30; NormBCS) or high BCS ≥ 3.75 (*n* = 19; HighBCS) groups. In HighBCS cows, parity (mean ± SD) prior to calving averaged 2.86 ± 1.15 while in NormBCS it averaged 3.15 ± 1.45. Previous 305-d milk yield was 10,742 ± 2016 kg for HighBCS cows and 11,080 ± 2031 kg for NormBCS cows. Cows in HighBCS averaged (mean ± SD) 51.80 ± 4.08 d dry and NormBCS cows averaged 50.05 ± 5.10 d dry. Sires used (6 bulls) on these cows were positive for production, health, and fertility traits. Body condition score was measured from − 4 to − 1 weeks prior to calving on a 5-point scale (1 = thin, 5 = fat) in increments of 0.25 point [17] and the average score for NormBCS cows was 3.02 ± 0.17 and the average for the HighBCS group was 3.83 ± 0.15. Body weight was measured using digital scale and BCS (scale 5) was taken by 2 people once a week. All cows received the same close-up diet starting from − 28 d of expected calving date (1.37 Mcal/kg of DM, 8.43% rumen degradable protein, and 6.13% rumen undegradable protein). Diet composition is available in Supplemental Table 1. During the dry period cows were housed in a sand bedded free-stall barn with access to feed through an individual Calan gate feeding system (American Calan, Northwood, NH, USA). When the expected calving date approached, cows were moved to individual pens bedded with straw.

### Animal management

In total, 49 calves born to dams with NormBCS (*n* = 30, 15 heifers and 15 bulls) or HighBCS (*n* = 19, 10 heifers and 9 bulls) were used. Immediately after birth calves were removed from their dam, and growth measurements were taken. All calves received 3.8 L of first colostrum from their respective dam, if voluntary intake of colostrum did not reach 3.8 L calves were force-fed via esophageal tube to reach the required amount of colostrum. All calves were managed in the same fashion. Outdoor individual hutches were used, and calves were fed with milk replacer (Advance Excelerate, Milk Specialties, Carpentersville, IL; 28.5% CP, 15% fat) (06:00 and 18:00 h) until 35 d of age. From 36 d of age until weaning (42 d of age) calves were switched to once a day feeding at 6:00 h. Calves received 4.54 kg/d of milk replacer (0.59 kg of milk replacer in 3.95 L of water) from 1 to 10 d of age, 5.90 kg/d (0.77 kg of milk replacer in 5.13 L of water) from 11 to 20 d of age, 7.26 kg/d (0.94 kg of milk replacer in 6.32 L of water) from 21 to 35 d of age, and 3.63 kg/d (0.47 kg of milk replacer in 3.16 L of water) in a single feeding from 36 to 42 d of age. Calves had ad libitum access to a starter grain mix (Ampli-Calf Starter 20®; 19.9% CP, 13.5% neutral detergent fiber; Purina Animal Nutrition, Shoreview, MN, United States) at 08:00 h from d 1 until d 65 of age. Starter intake was recorded daily and body weight with other growth measurements were taken weekly and used to calculate average daily gain.

### Sample collection

Individual feed ingredients and total mixed ration (TMR) samples were collected once a week and the ration was adjusted accordingly to maintain DM ratios of the ingredients. Weekly samples of ingredients and TMR were frozen at − 20 °C and composited monthly for nutrient composition analysis by standard wet chemistry techniques at a commercial laboratory (Dairy One, Ithaca, NY, USA).

Cow blood samples were collected from the coccygeal vein on d − 10 ± 2 relative to expected calving date. Calf blood samples were collected from the jugular vein at birth (before receiving colostrum, 0 d), 24 h after first colostrum and at 7, 21, 42 and 50 d of age. Plasma was obtained by centrifugation of sodium heparin tubes, at 1900×*g* for 15 min and stored at − 80 °C until further analysis. Aspartate aminotransferase (AST, catalog no. 0018257540), bilirubinn (catalog no. 0018254640), glucose (catalog no. 001825840), and urea (catalog no. 001825760), gamma-glutamyl transferase (GGT, catalog no. 0018254240) in plasma were measured with kits purchased from Instrumentation Laboratory Spa (Werfen Co., Milan, Italy). The method described by Skinner et al. [18] was used to measure haptoglobin and adapted to the ILAB 650 clinical auto-analyzer (Instrumentation Laboratory, Lexington, MA, USA). The method of Ferré et al. [19] was adapted to the ILAB 650 conditions to determine concentrations of paraoxonase. The d-ROMs-test (cod. MC002) purchased from Diacron (Grosseto, Italy) was used to measure reactive oxygen metabolites (ROM). The ferric-reducing antioxidant power (FRAP) was used to measure total antioxidant capacity using a published colorimetric method [20]. Activity of myeloperoxidase (MPO) was measured via colorimetry based on the reaction of MPO contained in the plasma sample with hydrogen peroxide as described previously [21]. Concentrations of total nitric oxide metabolites (NO_x_), nitrites, and nitrates were determined using the methods described by Trevisi et al. [22]. Retinol and tocopherol were extracted with hexane and analyzed by reverse-phase HPLC using Spherisorb ODS-2, 3 μm, in a 150 mm × 4.6 mm column (Alltech, Deerfield, IL, USA); a UV detector set at 325 (for retinol) or 290 nm (for tocopherol); and methanol:tetrahydrofuran = 80:20 as the mobile phase. Concentrations of nonesterified fatty acids (NEFA) and β-hydroxybutyrate (BHBA) were measured using kits from Wako Chemicals (Richmond, VA, USA) and Randox Laboratories Ltd. (Crumlin, United Kingdom), respectively. The inter-assay coefficients of variation ranged between 0.75% (glucose) to 5.00% (NEFA), and the intraassay coefficients of variation between 0.82% (BHBA) and 3.64% (haptoglobin).

### Statistical analysis

Data were analyzed with the Proc MIXED procedure of SAS 9.4 (SAS Institute Inc., Cary, NC, USA). Fixed effects in the model were maternal group (BCS), day (D), and their interaction (BCS × D). Random effect was calf within (maternal BCS). The covariance structure SP (POW) for repeated measures was used for the analysis of plasma data over times. The covariate structure (AR) (1) was used for the analysis of growth measurements and daily starter intake. Additionally, Pearson correlation coefficients between concentrations of blood variables prior to calving in cows and their offspring body weight (BW) at birth were calculated using PROC CORR of SAS. Data are presented as least-square means with pooled SEMs. Significance was declared at *P* ≤ 0.05.

## Results

### Cow performance and blood plasma biomarkers at − 10 d relative to parturition

**Fig. 1:**
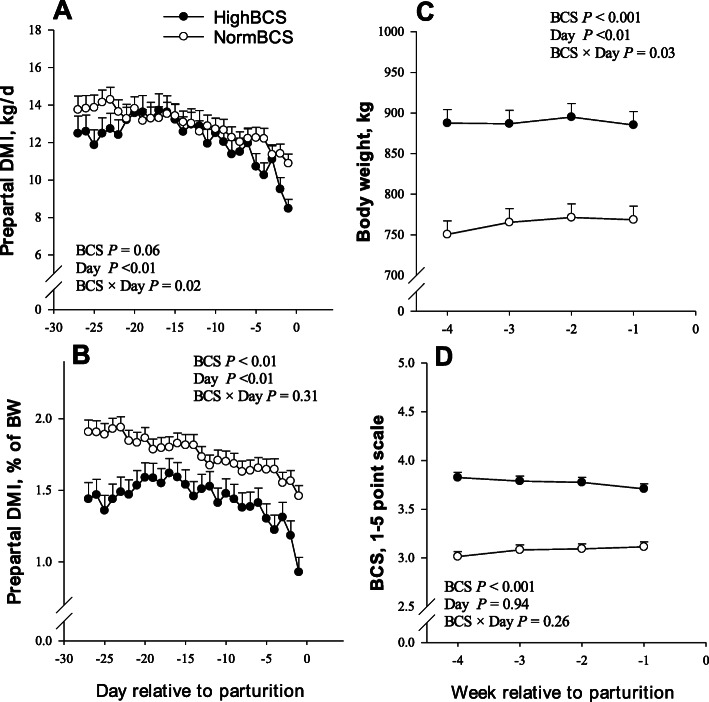
Daily dry matter intake (**a** and **b**), body weight (**c**), and body condition score (BCS; **d**) during late-pregancy in cows with a BCS ≤ 3.25 (NormBCS, **○**; *n* = 30) or BCS ≥ 3.75 (HighBCS, **●**; *n* = 19) at −4 weeks relative to parturition

Overall DMI expressed as kg/d or % of BW during the last 4 weeks of pregnancy was lower (*P* < 0.01;1.43 vs. 1.75 ± 0.07 DMI % of BW) (Fig. 1) for HighBCS cows. A significant interaction (BCS × day) was detected due to lower (*P* = 0.02) DMI during last 4 d of pregnancy in HighBCS cows. Prior to parturition, the HighBCS group had greater overall body weight (*P* < 0.01; 888 vs. 763 ± 20.4 kg) and BCS (*P* < 0.01; 3.77 vs. 3.08 ± 0.05) (Fig. 1).

In plasma, overall concentrations of glucose, NEFA, ceruloplasmin, and NO_x_ were greater and cholesterol and urea lower (*P* ≤ 0.05, Table 1) at − 10 d in HighBCS cows, while ROM tended (*P* = 0.08) to be greater in those cows.

**Table 1:** Concentrations of plasma biomarkers at − 10 d relative to parturition in cows classified according to normal body condition score (BCS) (≤ 3.25, NormBCS; *n* = 30) or high BCS (≥ 3.75, HighBCS; *n* = 19) at 4 weeks prior to parturition

Item^a^	Cow BCS		SEM	
	HighBCS	NormBCS	HighBCS	NormBCS
Energy metabolism				
BHBA, mmol/L	0.44	0.46	0.03	0.02
Creatinine, μmol/L	96.2	95.4	1.98	1.40
Glucose, mmol/L	4.59^*^	4.39	0.07	0.05
NEFA, mmol/L	0.30^*^	0.19	0.03	0.02
Urea, mmol/L	5.40^*^	6.16	0.19	0.26
Liver function				
AST/GOT, U/L	68.7	75.3	3.37	2.72
Cholesterol, mmol/L	2.54^*^	2.86	0.13	0.09
GGT, U/L	18.8	20.5	1.43	1.01
Total bilirubin, μmol/L	1.12	0.98	0.26	0.16
Inflammation				
Albumin, g/L	36.2	36.3	0.36	0.26
Ceruloplasmin, μmol/L	2.54^*^	2.30	0.09	0.07
Haptoglobin, g/L	0.26	0.26	0.03	0.02
Myeloperoxidase, U/L	478	453	30.8	20.9
Oxidative stress				
Paraoxonase, U/mL	86.9	87.7	4.09	2.89
FRAP, μmol/L	104	111	3.37	2.60
NO_x_, μmol/L	31.5^*^	29.9	0.64	0.47
Nitrites, μmol/L	7.51	7.24	0.67	0.47
Nitrates, μmol/L	23.3	22.4	0.82	0.58
ROM, mg H_2_O_2_/100 mL	13.3	12.5	0.37	0.27
Retinol, μg/100 mL	27.9	31.5	1.87	1.35
Tocopherol, μg/mL	3.49	3.81	0.34	0.24

^a^*AST* aspartate aminotransferase, *BHBA* betahydroxybutyrate, *GGT* γ-glutamyl transpeptidase, *FRAP* ferric reducing ability of plasma, *NEFA* nonesterified fatty acids, *NO*_*x*_ nitric oxide, *ROM* reactive oxygen metabolites

^*^Means differ *P ≤* 0.05 between groups

### Calf growth performance

In HighBCS cows, a negative correlation (Table 2) was observed between calf BW and maternal concentrations of haptoglobin (r = − 0.76; *P* < 0.01), ceruloplasmin (r = − 0. 57; *P* = 0.05), and ROM (r = − 0.79; *P* < 0.01) at − 10 d. In contrast, a positive correlation between urea (r = 0.55; *P* = 0.05) and BW at birth was detected in HighBCS calves.

**Table 2:** Pearson correlations between concentrations of cow plasma biomarkers at −10 d relative to parturition and their offspring body weight. Cows were classified according to normal body condition score (BCS) (≤ 3.25, NormBCS; *n* = 30) or high BCS (≥ 3.75, HighBCS; *n* = 19)

Item^a^	Cow BCS		*P*-value	
	HighBCS	NormBCS	HighBCS	NormBCS
Energy metabolism				
BHBA, mmol/L	0.30	−0.31	0.32	0.12
Creatinine, μmol/L	−0.03	< 0.001	0.91	0.99
Glucose, mmol/L	−0.02	−0.07	0.97	0.73
NEFA, mmol/L	0.01	−0.02	0.96	0.94
Urea, mmol/L	0.55	−0.27	0.05	0.18
Liver function				
AST/GOT, U/L	−0.46	−0.01	0.12	0.95
Cholesterol, mmol/L	−0.01	−0.07	0.99	0.98
GGT, U/L	−0.57	−0.11	0.03	0.58
Total bilirubin, μmol/L	0.02	−0.21	0.94	0.31
Inflammation				
Albumin, g/L	0.42	−0.32	0.15	0.11
Ceruloplasmin, μmol/L	−0.58	−0.03	0.03	0.88
Haptoglobin, g/L	−0.76	0.31	< 0.001	0.13
Myeloperoxidase, U/L	−0.41	0.09	0.16	0.64
Oxidative stress				
Paraoxonase, U/mL	0.23	−0.05	0.46	0.80
FRAP, μmol/L	−0.27	0.28	0.37	0.17
NO_x_, μmol/L	0.41	−0.05	0.16	0.81
Nitrites, μmol/L	0.01	0.26	0.96	0.20
Nitrates, μmol/L	0.41	−0.36	0.16	0.07
ROM, mg H_2_O_2_/100 mL	−0.73	0.11	< 0.005	0.61
Retinol, μg/100 mL	−0.05	0.05	0.88	0.82
Tocopherol, μg/mL	0.30	−0.14	0.32	0.49

^a^*AST* aspartate aminotransferase, *BHBA* betahydroxybutyrate, *GGT* γ-glutamyl transpeptidase, *FRAP* ferric reducing ability of plasma, *NEFA* nonesterified fatty acids, *NO*_*x*_ nitric oxide, *ROM* reactive oxygen metabolites

**Fig. 2:**
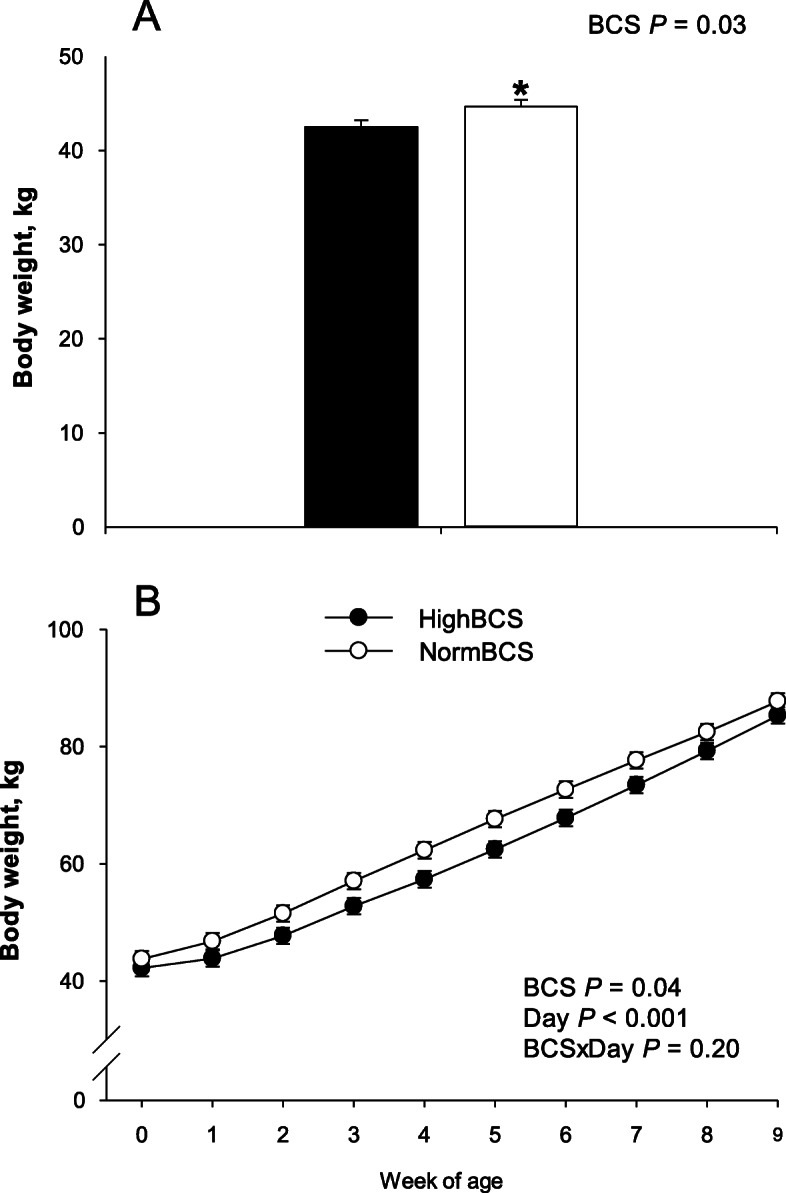
Body weight at birth (panel **a**) and through the first 9 weeks of age in calves born to cows with a body condition score (BCS) ≤ 3.25 (NormBCS, **○**; *n* = 30) or BCS ≥ 3.75 (HighBCS, **●**; *n* = 19) at −4 weeks relative to parturition

At birth, calves born to dams with HighBCS had lower BW (*P* = 0.03; 42.52 vs. 44.67 ± 0.72 kg) (Table 3, Fig. 2). Ratio of calf birth BW to cow BW at − 4 weeks from parturition also was lower in HighBCS calves (Table 3). Postnatally, BW in HighBCS calves remained lower through 9 weeks of age (*P* < 0.05; Table 4, Fig. 2). However, hip height, hip width, wither height, body length, daily starter intake and average daily gain did not differ due to maternal BCS (*P* > 0.10; Table 4).

**Table 3:** Growth measurements and concentrations of plasma biomarkers at birth in calves born to cows classified according to normal body condition score (BCS) (≤ 3.25, NormBCS; *n* = 30) or high BCS (≥ 3.75, HighBCS; *n* = 19) at 4 weeks prior to parturition

Item^a^	Maternal BCS		SEM		*P*-value
	HighBCS	NormBCS	HighBCS	NormBCS	
Body weight (BW), kg	42.5^*^	44. 7	0.72	0.63	0.03
Calf BW/Cow BW^b^	0.048	0.059	0.002	0.001	< 0.001
Hip height, cm	78.8	78.9	0.78	0.61	0.86
Hip width, cm	15.1	15.1	0.20	0.16	0.84
Wither height, cm	76.1	76.8	0.40	0.32	0.20
Body length, cm	111	110	1.0	1.2	0.49
Plasma biomarkers					
Energy metabolism					
BHBA, mmol/L	0.03^*^	0.05	0.006	0.004	0.01
Creatinine, μmol/L	170	179	7.20	4.56	0.32
Glucose, mmol/L	4.56	3.93	0.30	0.21	0.09
NEFA, mmol/L	0.77^*^	1.08	0.11	0.08	0.03
Urea, mmol/L	5.31^*^	5.97	0.24	0.19	0.04
Liver function					
AST/GOT, U/L	46.4	51.7	3.97	2.96	0.30
Cholesterol, mmol/L	0.58	0.64	0.05	0.04	0.32
GGT, U/L	10.5	9.93	1.08	0.83	0.70
Total bilirubin, μmol/L	10.6	13.5	1.17	0.90	0.06
Inflammation					
Albumin, g/L	28.9^*^	30.0	0.35	0.27	0.02
Ceruloplasmin, μmol/L	0.04^*^	0.10	0.02	0.01	0.01
Haptoglobin, g/L	0.33	0.33	0.02	0.02	0.76
Myeloperoxidase, U/L	217	198	20	16	0.47
Oxidative stress					
Paraoxonase, U/mL	9.71	9.43	2.21	1.89	0.92
FRAP, μmol/L	250	247	12.63	9.71	0.85
NO_x_, μmol/L	202	197	10.72	7.92	0.74
Nitrites, μmol/L	3.04	3.71	0.31	0.24	0.09
Nitrates, μmol/L	199	194	10.81	7.99	0.71
ROM, mg H_2_O_2_/100 mL	4.91	5.06	0.26	0.20	0.67
Retinol, μg/100 mL	8.40	8.43	0.78	0.58	0.97
Tocopherol, μg/mL	0.39	0.41	0.03	0.02	0.59

^a^*AST* aspartate aminotransferase, *BHBA* betahydroxybutyrate, *GGT* γ-glutamyl transpeptidase, *FRAP* ferric reducing ability of plasma, *NEFA* nonesterified fatty acids, *NO*_*x*_ nitric oxide, *ROM* reactive oxygen metabolites

^b^Ratio of calf birth BW to cow BW at −4 weeks relative to parturition

^*^Means differ *P* ≤ 0.05 between

### Calf plasma biomarkers

**Fig. 3:**
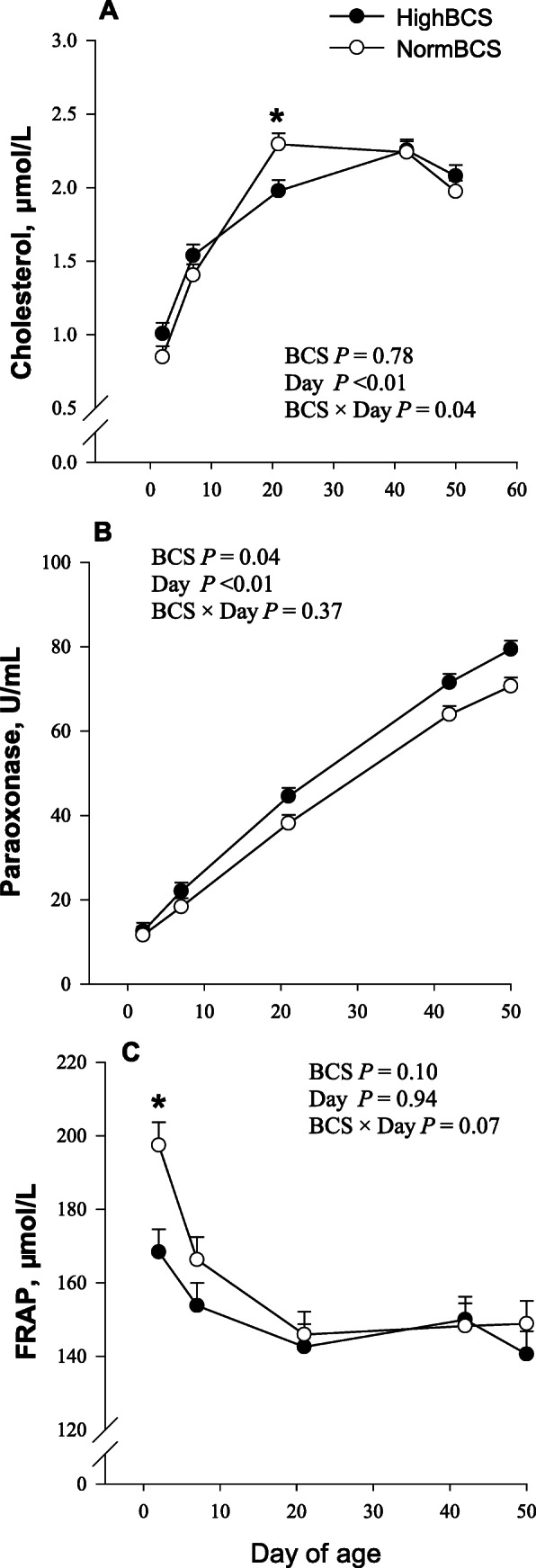
Plasma concentrations of cholesterol (**a**), activity of paraoxonase (**b**), and ferric reducing ability of plasma (FRAP; **c**) from birth through day 50 of age in calves born to cows with a body condition score (BCS) ≤ 3.25 (NormBCS, **○**; *n* = 30) or BCS ≥ 3.75 (HighBCS, **●**; *n* = 19) at − 4 weeks relative to parturition. Calves were weaned at 42 d of age

At birth, calves from HighBCS cows had lower (*P* < 0.05; Table 3) overall plasma concentrations of ceruloplasmin, albumin, urea, NEFA, and BHBA, while nitrite tended (*P* = 0.09) to be lower. Among the plasma biomarkers analyzed, total bilirubin and paraoxonase (Fig. 3) were affected by maternal BCS throughout the first 50 d of age; total bilirubin concentration was lower (*P* < 0.05; Table 4) and paraoxonase greater (*P* < 0.05) in HighBCS calves. Plasma FRAP also tended to be lower in HighBCS calves throughout the postnatal period evaluated (*P* = 0.07; Table 4; Fig. 3).

**Table 4:** Growth measurements and concentrations of plasma biomarkers during the first 50 d of age in calves born to cows classified according to normal body condition score (BCS) (≤ 3.25, NormBCS; *n* = 30) or high BCS (≥ 3.75, HighBCS; *n* = 19) at 4 weeks prior to parturition

Item^c^	Maternal BCS		SEM	*P*-value		
	HighBCS	NormBCS		BCS	Day	BCS × Day
Body weight, kg	59.6^*^	63.3	1.33	0.04	< 0.01	0.20
Hip height, cm	85.5	86.1	0.61	0.49	< 0.01	0.70
Hip width, cm	19.7	20.0	0.23	0.28	< 0.01	0.94
Wither height, cm	82.6	83.1	0.52	0.42	< 0.01	0.36
Body length, cm	124	125	1.0	0.86	< 0.01	0.46
Starter intake, kg/d	1.09	1.03	0.03	0.15	< 0.01	0.16
Average daily gain (ADG), kg/d	0.66	0.69	0.04	0.49	–	–
Plasma biomarkers						
Energy metabolism						
BHBA, mmol/L	0.10	0.09	0.01	0.13	< 0.01	0.25
Creatinine, μmol/L	89.7	91.9	1.75	0.33	< 0.01	0.34
Glucose, mmol/L	6.52	6.48	0.17	0.85	< 0.01	0.63
NEFA, mmol/L	0.17	0.17	0.01	0.94	< 0.01	0.66
Urea, mmol/L	4.43	4.52	0.11	0.53	< 0.01	0.06
Liver function						
AST/GOT, U/L	74.7	71.2	2.08	0.17	< 0.01	0.26
Cholesterol, mmol/L	1.75^*^	1.77	0.05	0.78	< 0.01	0.04
GGT, U/L	86.5	96.6	8.11	0.35	< 0.01	0.24
Total bilirubin, μmol/L	3.42^*^	4.22	0.22	0.01	< 0.01	0.11
Inflammation						
Albumin, g/L	31.4	34.5	0.36	0.89	< 0.01	0.23
Ceruloplasmin, μmol/L	2.32	2.39	0.11	0.64	< 0.01	0.52
Haptoglobin, g/L	0.32	0.33	0.02	0.59	< 0.01	0.06
Myeloperoxidase, U/L	310	340	20.19	0.26	< 0.01	0.90
Oxidative stress						
Paraoxonase, U/mL	46.0^*^	40.5	2.02	0.04	< 0.01	0.37
FRAP, μmol/L	150	160	4.30	0.10	< 0.01	0.07
NO_x_, μmol/L	84.7	85.8	7.44	0.91	< 0.01	0.94
Nitrites, μmol/L	5.07	5.10	0.33	0.94	< 0.01	0.09
Nitrates, μmol/L	76.2	78.8	7.52	0.79	< 0.01	0.97
ROM, mg H_2_O_2_/100 mL	12.0	11.9	0.48	0.86	< 0.01	0.38
Retinol, μg/100 mL	16.1	15.3	0.83	0.47	< 0.01	0.44
Tocopherol, μg/mL	2.73^*^	2.55	0.20	0.50	< 0.01	0.03

^c^*AST* aspartate aminotransferase, *BHBA* betahydroxybutyrate, *GGT* γ-glutamyl transpeptidase, *FRAP* ferric reducing ability of plasma, *NEFA* nonesterified fatty acids, *NO*_*x*_ nitric oxide, *ROM* reactive oxygen metabolites

^*^Means differ *P* < 0.05 between groups due to BCS or BCS×Day

## Discussion

### Cow performance and plasma variables

It has long been established that BCS around calving is negatively associated with DMI [1], thus, the lower DMI in HighBCS cows in the present study during the last 10 d of gestation was not unexpected. Further, compared with cows with a BCS > 3.5 during the last 4 weeks prepartum, cows with BCS < 3.17 had greater DMI as a percentage of BW prior to calving [23]. Adipose tissue plays an important role in the regulation of DMI and cows with high BCS mobilize more adipose tissue, putting them at greater risk to develop metabolic disorders such as fatty liver and subclinical ketosis [1, 2]. In accordance with this, HighBCS cows in the present study had greater circulating concentrations of NEFA, indicating an upregulation in lipolysis compared with NormBCS cows. However, NormBCS cows had greater circulating concentrations of urea. Urea is produced during the deamination of amino acids. Thus, the increase in plasma urea concentrations in NormBCS cows could represent a greater metabolism of amino acids and may reflect differences in DMI and ruminal ammonia synthesis between BCS groups.

Along with greater risk of developing metabolic disorders, cows with high BCS during the transition period are more likely to experience greater levels of oxidative stress and inflammation and reduced liver functionality [2, 24]. Ceruloplasmin is a positive acute-phase protein that is key for maintenance of iron homeostasis [25], and its production increases during periods of infection and inflammation, making it a good marker of inflammation in transition dairy cows [21]. The observed increases in plasma ceruloplasmin in the present study suggested that HighBCS cows experienced greater levels of inflammation than NormBCS cows. With regards to oxidative stress, a commonly measured variable is ROM. When ROM production increases, oxidative stress is induced and results in inflammatory responses and increased risk of metabolic disorders [26]. The tendency for an increase in plasma ROM in HighBCS cows is consistent with previous work reporting that cows with a high BCS are more sensitive to oxidative stress [2, 27]. The increase in ROM in the present study was likely related to the increased circulating NEFA in HighBCS cows, which could affected liver metabolism [28].

One of the commonly-used blood variables of liver function in dairy cattle is cholesterol [21, 26], which is required for the synthesis of lipoproteins [29]. Although the lower DMI in HighBCS cows partly explained the lower plasma cholesterol, it also suggested that the ability to synthesize and export lipoproteins might have been compromised. From a physiological standpoint, this effect might have rendered those cows more prone to developing fatty liver [30]. This hypothesis is also supported by the differences in circulating NEFA concentrations between both BCS groups. Overall, while the mechanisms could not be fully elucidated, the changes in plasma variables (greater NEFA, ceruloplasmin, and NO_x_; lower cholesterol) prior to calving suggested that HighBCS cows might have experienced more pronounced inflammation, oxidative stress and lower liver function [2, 4, 10, 24]. Furthermore, as reported previously [10], the negative correlations between maternal inflammation- and oxidative stress-related variables and birth BW in HighBCS calves underscored a physiological link, i.e. physiological stress in the cow during latter stages of gestation impacts the developing calf.

### Calf performance and plasma biomarkers

In non-ruminant species, it is well-documented that maternal body condition, e.g. obesity and starvation, alter offspring size and growth. Specifically, obese women are more likely to give birth to “large-for-gestational age” babies that will have an increased risk of becoming obese later in life [7]. In ruminants, effects of maternal body condition on offspring growth have primarily been explored in sheep and beef cattle. For instance, researchers working with pregnant ewes with low BCS of 2 or BCS > 3 (5-point scale) did not observe differences in lamb birth weight or postnatal growth [31]. Using the same division of maternal BCS, researchers also did not observe differences in lamb birth weight or growth through weaning and into adulthood (up to 2.5 years) [32]. Feeding beef cows to achieve a thin or moderate BCS from d 145 to d 260 of gestation did not alter fetal weight or length [33]. In another study, birth weight, ADG and average BW of beef calves through 60 d of age did not differ when their dams were fed to achieve a BCS of 4 or 6 at parturition (9-point scale) [34].

The greater birth weight and BW throughout the pre-weaning period in NormBCS vs. HighBCS calves could, at least in part, be explained by the greater DMI of NormBCS cows during the last 4 weeks of gestation. Maternal diets could alter placental transport of nutrients both in non-ruminant and ruminant species [35, 36]. Thus, greater intakes of digestible nutrients by NormBCS cows in the present study may have upregulated placental nutrient transport, leading to increased availability of nutrients for fetal growth. However, an average difference in maternal DMI of +0.84 kg/d prepartum by NormBCS cowsdid not fully account for the greater BW of their calves at birth (+2.2. kg on average) and through the period immediately postweaning (+3.7 kg on average).

Besides increased nutrient availability to the fetus and newborn, efficiency of nutrient utilization may have also been altered by maternal BCS. For instance, the greater concentrations of NEFA and BHBA at birth in calves born to NormBCS dams suggested they potentially had a more mature metabolism capable to readily use fat depots as a source of fatty acids for oxidation and ketogenesis. This hypothesis agrees with previous work indicating that neonates need to quickly activate processes such as lipolysis, glycogenolysis and gluconeogenesis to maintain normal blood glucose concentrations [37].

It has been documented in dairy calves that lactate and amino acids (AA) account for 60% of gluconeogenesis during the first 3 d of life [38]. Thus, a greater reliance on fatty acids for energy in NormBCS calves seems likely in part due to a greater usage of other nutrients such as AA for gluconeogenesis. This idea is supported by the fact that plasma urea concentrations were greater in NormBCS calves at birth. Because plasma glucogenic AA concentrations were not measured to support this hypothesis, future studies should consider such analysis to better understand how AA utilization for gluconeogenesis is altered by maternal BCS during late-gestation.

Albumin is a negative acute-phase protein that is decreased during periods of inflammation [26]. The greater plasma concentrations of albumin at birth in calves born to NormBCS suggested that levels of inflammation were lower. Such a difference in the degree of inflammation has also been observed in rodent and human studies where maternal obesity was associated with increased fetal and offspring inflammation [39].

With regards to immune response, macrophages produce NO_x_ as a major mechanism to kill bacterial pathogens [40]. The nitric oxide that is produced can then be metabolized to both nitrites and nitrates [41]. In the present study, the tendency for greater nitrite concentrations in NormBCS calves at birth suggested that more NO_x_ was being produced and metabolized, suggesting that those calves had a better functioning (albeit still immature) immune system [42].

Ferric reducing ability of plasma (FRAP) is an important indicator of antioxidant power, as it is a measure of the reduction of ferric to ferrous ion [20]. Thus, the tendency for greater FRAP throughout the first 9 weeks of age in NormBCS calves suggested a better antioxidant response and potentially lower oxidative stress. A similar response has been observed in non-ruminants, where maternal obesity was associated with an increase in fetal and offspring oxidative stress [43]. Furthermore, the negative correlations between concentrations of haptoglobin, ROM and ceruloplasmin in HighBCS cows with calf birth weight not only suggested that greater levels of inflammation related to overconditioning have a negative impact on fetal growth, but also postnatal development. This idea is further supported by the fact that HighBCS calves did not reach the same BW as NormBCS until approximately 8–9 weeks of age.

In addition to the temporal changes in growth, it is noteworthy to highlight the few differences in the concentrations of plasma variables of liver function throughout the first 9 weeks of life. For instance, paraoxonase is an enzyme produced by the liver that associates with high-density lipoproteins in blood to prevent oxidation [19]. Bilirubin is produced during heme degradation and plasma concentrations increase when liver function is impaired due to reduced clearance. Although the greater overall plasma bilirubin in NormBCS calves could be taken as a sign they experienced decreased liver function, these calves tended to have greater concentrations already at birth, which agrees with the notion they were born with a more mature metabolism. The effects of maternal BCS during late-gestation on offspring liver function have not been studied in dairy cattle. However, data from human and rodent studies with increased maternal body mass index indicated a negative association of that trait with risk of fatty liver disease and liver function in the offspring [44].

As discussed earlier, changes in plasma NEFA and BHBA concentrations at birth suggested that NormBCS calves were utilizing more fatty acids a source of energy. While increased mitochondrial oxidation of fatty acids would be expected to improve liver function due to the reduction in fat storage in the liver, it would also enhance the production of reactive oxygen species. Clearly, further research could help better understand the physiological mechanisms that may be affected in the neonate as a result of high maternal body condition.

## Summary and conclusions

Overall, calves born from dams with higher than normal BCS (3.83 ± 0.15) during the last ~ 4 weeks prepartum had lower BW at birth and throughout the first 9 weeks of life. At the whole-animal level these effects did not seem to be fully associated with differences in maternal DMI or feed intake by the calves. Thus, alterations in inflammatory and oxidative stress status along with nutrient utilization in utero likely played a role. Whether these encompass “programming” effects at the cellular level could not be discerned, but based on non-ruminant data it appears likely. Additional work clearly is needed to clarify the mechanisms by which maternal BCS along with other factors such as genetics, calving season, and previous lactation regulate immunometabolism in the developing fetus and neonatal calf.

## Supplementary Information

**Additional file 1: Supplementary Table 1.** Ingredient and nutrient composition of the diet fed during close-up dry period (−28 d to calving)

## Data Availability

The data reported in this manuscript is available upon reasonable request from the corresponding author (JJL).

## Abbreviations

AST: Aspartate aminotransferase
BCS: Body condition score
BHBA: Betahydroxybutyrate
BW: Body weight
DMI: Dry matter intake
FRAP: Ferric-reducing antioxidant power
GGT: Gamma-glutamyl transferase
HighBCS: High body condition score
MPO: Myeloperoxidase
NEFA: Nonesterified fatty acids
NormBCS: Normal body condition score
NO_x_: Nitric oxide
ROM: Reactive oxygen metabolites
TMR: Total mixed ration
