# Supplementary material for: Maternal body condition during late-pregnancy is associated with in utero development and neonatal growth of Holstein calves
Source: J Anim Sci Biotechnol. 2021 Apr 2;12:44. doi: 10.1186/s40104-021-00566-2 (PMC8017770; doi:10.1186/s40104-021-00566-2)
Supplement: Supplementary file 1 — Additional file 1: Supplementary Table 1. Ingredient and nutrient composition of the diet fed during close-up dry period (−28 d to calving) [file 40104_2021_566_MOESM1_ESM.docx]

| **Supplemental Table 1**. Ingredient and nutrient composition of the diet fed during close-up dry period (−28 d to calving). | | |
| --- | --- | --- |
| Item | Content |  |
| Ingredient, % of DM |  |  |
| Corn silage | 37.47 |  |
| Ground shelled corn | 11.60 |  |
| Wheat straw | 21.82 |  |
| Canola meal | 11.67 |  |
| Soybean meal | 6.30 |  |
| Alfalfa hay | - |  |
| Soychlor^1^ | 3.37 |  |
| Corn gluten feed | 2.80 |  |
| ProvAAL2 AADvantage^2^ | 0.47 |  |
| Biotin^3^ | 0.10 |  |
| Rumensin^4^ | 0.19 |  |
| Calcium sulfate | 0.53 |  |
| Magnesium oxide | 0.10 |  |
| Ca | 0.66 |  |
| P | 0.33 |  |
| Salt | 0.10 |  |
| Na | 0.12 |  |
| Cl | 0.78 |  |
| Mg | 0.45 |  |
| K | 1.36 |  |
| S | 0.33 |  |
| Nutrient composition |  |  |
| CP, % of DM | 14.50 |  |
| NDF, % of DM | 43.30 |  |
| ADF, % of DM | 33.80 |  |
| NFC, % of DM | 28.22 |  |
| NE_L_, Mcal/kg of DM | 1.37 |  |
| NE_L_ allowable milk, kg/d | - |  |
| MP allowable milk, kg/d | - |  |
| RDP, % of DM | 8.45 |  |
| RUP, % of DM | 6.05 |  |
| RDP required, g/d | 1,165 |  |
| RDP supplied, g/d | 1,152 |  |
| RDP balance, g/d | -18 |  |
| RUP required, g/d | 158 |  |
| RUP supplied, g/d | 821 |  |
| RUP balance, g/d | 662 |  |
| MP required, g/d | 821 |  |
| MP supplied, g/d | 1,360 |  |
| MP balance, g/d | 539 |  |
| ^1^West Central Soy.  ^2^Perdue AgriBusiness (Salisbury, MD).  ^3^ADM Animal Nutrition (Quincy, IL).  ^4^Rumensin, Elanco Animal Health (Greenfield, IN). | | |
